# Psychological Characteristics of Mothers of Children with Chronic Illnesses: A Focus on Type 1 Diabetes Mellitus

**DOI:** 10.3390/healthcare13121439

**Published:** 2025-06-16

**Authors:** Eleni Albani, Elena Dragioti, Konstantina Dimou, Stefanos Mantzoukas, Mary Gouva

**Affiliations:** Research Laboratory Psychology of Patients, Families and Health Professionals, School of Health Sciences, University of Ioannina, 45110 Ioannina, Greece; ealbani@upatras.gr (E.A.); dragioti@uoi.gr (E.D.); dimou@uoi.gr (K.D.); smantzoukas@uoi.gr (S.M.)

**Keywords:** Type 1 Diabetes Mellitus (T1DM), maternal mental health, spirituality, somatization, psychological distress, caregiver burden

## Abstract

**Background:** Mothers of children with Type 1 Diabetes Mellitus (T1DM) frequently face high levels of psychological stress. While the impact of this stress on caregiving is well documented, the potential role of spirituality as a protective factor has received limited attention. **Objective:** This cross-sectional study aimed to assess the relationships among psychological symptoms, spirituality, and coping in mothers caring for children with T1DM, with a particular focus on the potential protective role of spirituality in caregiver resilience. **Methods:** A total of 134 mothers completed validated Greek versions of the Symptom Checklist-90 (SCL-90) and the Spiritual and Religious Attitudes in Dealing with Illness (SpREUK) questionnaire. Descriptive statistics, Pearson correlations, and multiple linear regression analyses were used to explore associations and predictive relationships. **Results:** Participants reported elevated psychological symptoms, particularly somatization, anxiety, and depression. Regression analysis revealed that trust in higher guidance was a significant negative predictor of somatization (b = −0.775, *p* < 0.001), indicating a potential buffering effect. In contrast, the search for support was positively associated with somatization (b = 0.704, *p* < 0.001), suggesting a more reactive coping pattern. Other spiritual variables, including the total spirituality score and reflection, were not statistically significant in the multivariate model. **Conclusions:** These findings highlight the relevance of spirituality—particularly spiritual trust—in understanding psychological distress among caregiving mothers. Incorporating existential and spiritual elements into psychosocial interventions may enhance maternal well-being. Longitudinal studies are needed to clarify spirituality’s role as a protective or mediating factor in chronic caregiving contexts.

## 1. Introduction

Caring for a child with a chronic illness, such as Type 1 Diabetes Mellitus (T1DM), constitutes a profound source of psychological stress for parents, with mothers in particular experiencing a disproportionate burden due to prevailing cultural expectations and their predominant role as primary caregivers [1,2]. This enduring responsibility frequently results in heightened emotional and psychological strain, manifesting in a variety of adverse outcomes.

A substantial body of evidence underscores the critical role of spirituality in promoting psychological resilience among individuals confronting chronic adversity [3,4,5]. Spirituality has been posited to endow individuals with a sense of meaning, hope, and inner strength, thereby facilitating more adaptive coping responses [5]. Within the context of T1DM, mothers are routinely tasked with the complex and relentless management of their child’s illness, encompassing insulin administration, rigorous blood glucose monitoring, and dietary regulation. These multifaceted demands intensify psychological stress and have demonstrable repercussions for both maternal mental health and broader family dynamics [6].

The prevalence of psychological distress among mothers of children with T1DM is well documented. Empirical studies have identified elevated rates of anxiety, depression, and symptomatology akin to post-traumatic stress disorder (PTSD) within this population. A comprehensive systematic review indicated that approximately 33.5% of parents reported significant psychological distress at the time of diagnosis, with 19% experiencing persistent distress between one and four years post-diagnosis [7]. This enduring psychological burden has both direct and indirect implications for pediatric disease management; chronic distress in mothers has been associated with poorer glycemic control in children and suboptimal adherence to treatment protocols [8].

Moreover, the psychological sequelae experienced by mothers are multifactorial. Beyond the overt medical and logistical demands, mothers must continually provide emotional support to their child, contributing to a persistent risk of caregiver burnout. Burnout in this context is characterized by emotional exhaustion, diminished self-efficacy, and an increased propensity for family conflict [2]. These dynamics often perpetuate a cycle wherein maternal psychological distress further exacerbates difficulties in pediatric disease management, as evidenced by correlations between elevated parental stress, family discord, and increased HbA1c levels [8].

Sociocultural factors further exacerbate maternal stress, particularly in societies where caregiving responsibilities are predominantly ascribed to mothers [6]. The initial response to a child’s diagnosis commonly encompasses shock and disbelief, succeeded by chronic stress as mothers adapt to the complexities of their caregiving role [7]. The empirical literature suggests that the availability of social support networks and culturally attuned interventions may attenuate these stressors and foster greater resilience [6,7].

Intervention programs targeting maternal psychological distress have demonstrated efficacy in enhancing coping capacity and reducing perceived isolation. These programs typically integrate education regarding diabetes management, access to psychological counseling, and participation in peer support groups. Furthermore, systematic screening for depression and anxiety is recommended for the early identification and support of mothers at risk [7]. The adoption of a holistic healthcare paradigm that addresses both medical and psychological dimensions is considered essential for optimizing outcomes in families affected by chronic pediatric conditions.

The psychological well-being of mothers thus emerges as a critical determinant of both effective disease management and overall family functioning [9,10]. Evidence suggests that comprehensive support systems and targeted interventions addressing maternal mental health can yield substantial benefits for both mothers and their children. Accordingly, continued research and policy initiatives should prioritize the integration of psychological care into standard pediatric chronic illness management protocols. Recognizing the pivotal role of maternal psychological health in disease management and family well-being, and the imperative to inform evidence-based interventions, it is essential to identify the psychosocial resources that underpin resilience in this population. Within this framework, the present study addresses a critical gap by investigating the specific contribution of spirituality to psychological adaptation in mothers caring for children with T1DM.

### Aim of the Study

The principal aim of this study was to investigate the association between spirituality and psychological symptoms—including somatization, anxiety, guilt, aggression, and paranoid ideation—in mothers of children with T1DM. Beyond simply describing levels of distress or coping, the study sought to elucidate whether specific spiritual dimensions serve as protective factors or moderators of psychological distress and to evaluate their predictive value within a multivariate framework. In doing so, this research positions spirituality as a potential resource for resilience in the face of chronic caregiving demands.

## 2. Methods

### 2.1. Participants and Study Design

This study employed a cross-sectional design to elucidate psychological and spiritual profiles among mothers of children with T1DM in Greece. Between July and August 2024, a total of 150 mothers were recruited from pediatric diabetes clinics affiliated with university hospitals and community health centers nationwide. Recruitment was carried out by trained research assistants and clinicians working at participating pediatric diabetes clinics and community health centers. Informational brochures were distributed, and eligible mothers were approached during clinic visits or contacted via telephone following referral by healthcare staff.

Inclusion criteria stipulated biological motherhood of a child diagnosed with T1DM, primary caregiving responsibility, fluency in the Greek language, and informed consent to participate. Exclusion criteria encompassed incomplete demographic or clinical records, a self-reported history of severe psychiatric disorders (notably psychosis or bipolar disorder), or concurrent engagement in intensive psychotherapy. After rigorous screening procedures and the exclusion of 16 participants due to missing data, the final analytic cohort comprised 134 mothers. This sample size is consistent with standards for multivariate analysis in psychosocial research, which typically require a minimum of 10–15 participants per predictor variable to ensure adequate statistical power and model stability [11,12].

### 2.2. Measurements

Data collection entailed both sociodemographic and standardized psychometric instruments. Participants first completed an anonymous questionnaire capturing relevant personal characteristics and caregiving context. Collected sociodemographic variables included maternal age (in years), number of children, age of the child diagnosed with T1DM (in years), maternal education level (elementary, middle school, high school, or university), and marital status (married, divorced, widowed, or never married). Additionally, the primary disease-related variable was the number of years since the child’s diagnosis with T1DM.

Psychological symptomatology was assessed using the validated Greek version of the Symptom Checklist-90 (SCL-90) [13]. The SCL-90 is a widely used self-report inventory consisting of 90 items, designed to evaluate psychological distress and symptoms across nine domains: somatization, obsessive–compulsive symptoms, interpersonal sensitivity, depression, anxiety, hostility, phobic anxiety, paranoid ideation, and psychoticism. Each item is rated by respondents on a five-point Likert scale ranging from 0 (“not at all”) to 4 (“extremely”), reflecting the extent to which each symptom has been experienced in the past week [13,14,15]. The SCL-90 yields subscale scores for each domain as well as three global indices: the Global Severity Index (GSI), which represents overall psychological distress; the Positive Symptom Distress Index (PSDI), which reflects the intensity of reported symptoms; and the Positive Symptom Total (PST), which indicates the total number of symptoms endorsed at any level. The SCL-90 subscale scores represent symptom severity, with higher scores indicating greater psychological distress [13,14,15]. For the present study, analytic emphasis was placed on the nine primary symptom domains. The Greek version of the SCL-90 has demonstrated good psychometric properties in prior studies [13], with Cronbach’s alpha coefficients for the subscales in our sample ranging from 0.77 to 0.92, indicating acceptable to excellent internal consistency.

Spirituality and religious coping were assessed using the validated Greek version of the SpREUK questionnaire (Spiritual and Religious Attitudes in Dealing with Illness) [16]. The SpREUK is a multidimensional instrument originally developed to measure spirituality and religiosity in patients facing chronic illness [3,4,17], but it has been increasingly applied in other populations to assess the relevance of spiritual and religious beliefs and practices in coping with ill-related stressors [16,18]. The SpREUK comprises 15 items organized into three subscales: (1) search (for support/access), (2) trust (in higher guidance/source), and (3) reflection (positive interpretation of illness). Respondents rate each item on a five-point Likert scale from 1 (“does not apply at all”) to 5 (“applies very much”), with higher scores indicating stronger spiritual/religious attitudes [3,4,17,18]. Subscale scores are calculated as the mean of the corresponding items, where higher scores suggest stronger engagement with each spiritual dimension. Both subscales and a composite spirituality score, which was the sum of the three subscale scores, were computed in the current analysis [16]. The SpREUK has been validated in the Greek population [16], with Cronbach’s alpha coefficients for the subscales in our sample ranging from 0.71 to 0.96.

Hence, spirituality in this study refers to existential meaning, trust in higher guidance, and spiritual support seeking, as measured by the SpREUK questionnaire [3,4,17,18]. Notably, the SpREUK is distinguished by its avoidance of prescriptive religious language, rendering it suitable for respondents across a spectrum of belief systems and spiritual identities [17]. The application of the SpREUK in the current caregiver sample is justified by the growing body of evidence highlighting the significance of spirituality and religious coping in family members managing chronic illness, as these constructs may buffer psychological distress and support adaptive coping strategies [19,20].

### 2.3. Statistical Analysis

Missing data were handled by listwise deletion; 16 cases with incomplete demographic or instrument data were excluded. First, descriptive statistics were computed to summarize the primary spiritual dimensions, such as total spirituality, and the principal psychological variables based on SCL-90 subscale scores. The distribution of psychological scale scores was assessed using Shapiro–Wilk tests and Q-Q plots. Most psychological variables, including somatization, anxiety, and depression, approximated normal distribution, justifying the use of means and standard deviations for descriptive reporting. However, the 25th, 50th (median), and 75th percentiles, along with the ranges, for the SCL-90 and SpREUK subscales were also reported. In addition, key demographic characteristics, such as participants’ age, educational attainment, marital status, number of children, and years since the child’s diagnosis, were described using standard descriptive statistics and frequency distributions. Cronbach’s alpha coefficients were calculated for the internal consistency of each scale/subscale. Psychological and spiritual symptoms symptom scores were compared across groups defined by the duration of the child’s illness (≤2 years, 3–5 years, >5 years) using one-way analysis of variance (ANOVA). Subsequently, Pearson correlation coefficients were computed to examine associations among psychological, spiritual, and demographic variables. Special attention was given to the relationship between spirituality and somatization. Based on the observed correlations, a multiple linear regression analysis was then conducted to further explore predictors of somatization. In this analysis, somatization was specified as the dependent variable. The following psychological and spiritual variables were included as independent variables in the regression equation: obsessive–compulsive symptoms, interpersonal sensitivity, depression, anxiety, aggression, phobic anxiety, paranoid ideation, psychoticism, total spirituality score, search for support/access, trust in higher guidance/source, and reflection. While mothers’ age, education, marital status, and duration of the child’s illness were considered potential confounders, only psychological and spiritual variables were retained in the final regression model due to sample size constraints and collinearity diagnostics. Both unstandardized (B) and standardized beta coefficients and regression coefficients, as well as their corresponding *p*-values, were reported. Statistical significance was defined as *p* < 0.05. All findings, including descriptive statistics, correlation coefficients, and regression results, are presented in summary tables.

## 3. Results

### 3.1. Sample Demographics

Table 1 provides a detailed summary of the descriptive statistics for the demographic and psychological variables assessed in this study. In terms of disease-related experience, the average number of years since the child’s diagnosis with T1DM was 3.75 (SD = 2.07), with a range of 1 to 12 years. The children’s ages ranged from 1 to 17 years. This reflects a heterogeneous sample in terms of duration of exposure to the challenges of chronic caregiving—ranging from relatively recent diagnoses to long-standing caregiving experience.

The participating mothers had an average age of 39.62 years (SD = 5.28), with the youngest being 18 and the oldest 56 years old. This indicates a relatively mature sample, consistent with the life stage of mothers actively engaged in raising children. On average, mothers had 1.71 children (SD = 0.64), with the number of children ranging from one to three, suggesting a typical family structure in contemporary Greek households.

A majority of mothers were high school graduates (n = 130), comprising the largest educational subgroup. Another substantial portion of the sample had attained university-level education (n = 60), reflecting a well-educated cohort. Smaller segments included middle school graduates (n = 6) and elementary school graduates (n = 2). This educational distribution indicates that the majority of the sample has the cognitive and informational capacity to engage with complex health information, potentially influencing their coping mechanisms and caregiving strategies. The overwhelming majority of mothers were married (n = 182), highlighting the presence of a two-parent household in most cases. A smaller number reported being divorced (n = 13), widowed (n = 2), or never married (n = 1). This marital status distribution is important in contextualizing psychological outcomes, as the existing literature suggests that partnered mothers often have access to more emotional and practical support—factors that can mediate the psychological burden of caregiving.

Scores for both psychological and spirituality-related variables in this sample displayed substantial variability, highlighting a wide spectrum of experiences among participating mothers. For the psychological subscales (SCL-90), mean scores ranged from 2.66 (phobic anxiety) to 11.39 (depression), with all subscales showing minimum values of zero—indicating that some mothers reported no symptoms in certain domains. Maximum values for these subscales were notably high, with depression reaching up to 47 and obsessive–compulsive symptoms up to 32, suggesting that a subset of mothers experienced considerable psychological distress. Standard deviations were large relative to the means for all subscales, further illustrating the diversity in psychological symptom burden within the sample. For example, somatization had a mean of 6.91 (SD = 6.48), obsessive–compulsive symptoms 9.25 (SD = 5.98), and depression 11.39 (SD = 8.22).

Similarly, the spirituality variables demonstrated a broad range of scores. The total spirituality score averaged 41.61 (SD = 16.19) with a possible range of 0 to 75, reflecting considerable variation in spiritual attitudes and practices. Subscales such as search for support/access (mean = 11.87, SD = 7.30), trust in higher guidance/source (mean = 13.33, SD = 6.03), and reflection (mean = 16.42, SD = 4.33) also spanned the full range from 0 to 25. This pattern suggests that while some mothers reported minimal engagement with spirituality, others reported a high degree of spiritual involvement.

To further characterize the distribution of responses for the SCL-90 and SpREUK subscales, the 25th, 50th (median), and 75th percentiles for each subscale are reported in Supplementary Table S1. For somatization, the 25th, 50th, and 75th percentiles were 5, 5, and 8, respectively. For obsessive–compulsive, these values were 7, 9, and 10. For interpersonal sensitivity, percentiles were 2, 7, and 8; for depression, 6, 12, and 14; for anxiety, 3, 6, and 10; for aggressiveness, 1, 2, and 5; for phobic anxiety, 0, 2, and 4; for paranoid ideation, 4, 4, and 8; and for psychoticism, 1, 5, and 6. For total spirituality score, the 25th, 50th, and 75th percentiles were 36, 43, and 50, respectively. For search for support/access, these values were 7, 12, and 17. For trust in higher guidance/source, the percentiles were 9, 13, and 17; and for reflection, they were 13, 17, and 19.

**Figure 1:**
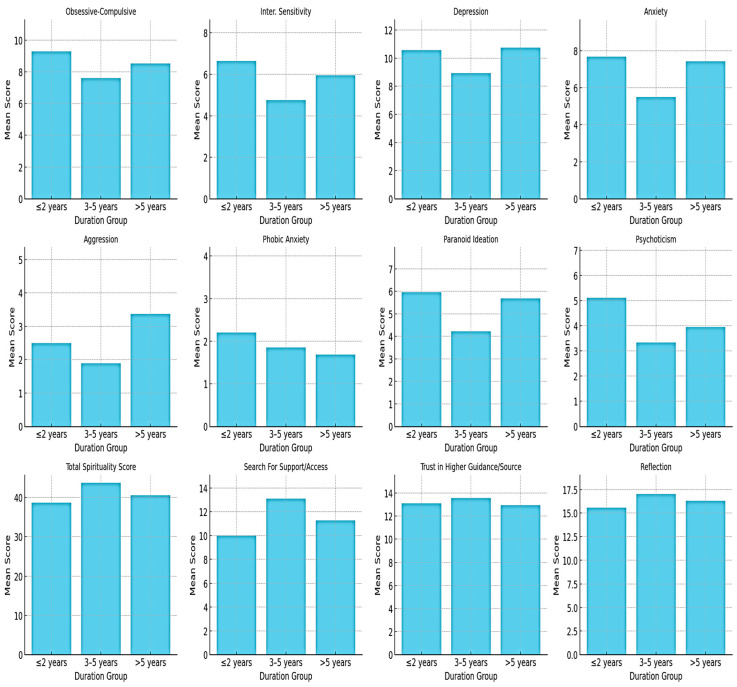
Group differences by duration of child’s illness.

Based on ANOVA, significant group differences (Figure 1) were found for interpersonal sensitivity (*p* = 0.015), anxiety (*p* = 0.012), aggression (*p* = 0.004), paranoid ideation (*p* = 0.005), and psychoticism (*p* = 0.006), with mothers in the shortest and longest duration groups reporting higher scores than those in the mid-duration group. In contrast, no statistically significant differences were observed for the total spirituality score or for any of the spirituality subscales across the duration groups (all *p* > 0.05).

### 3.2. Spiritual and Religious Attitudes and Psychopathology

The psychometric instruments employed in this study demonstrated acceptable internal consistency, as indicated by Cronbach’s alpha coefficients (Table 2) [21]. The correlation analysis revealed substantial associations among psychological variables. According to the adopted criteria, correlations above 0.70 were considered strong, those between 0.40 and 0.69 moderate, and those below 0.40 weak. Particularly strong relationships were observed between depression and obsessive–compulsive symptoms (r = 0.87, *p* < 0.001), anxiety and psychoticism (r = 0.84, *p* < 0.001), and interpersonal sensitivity and paranoid ideation (r = 0.86, *p* < 0.001). In contrast, spirituality variables demonstrated negative correlations with several psychological symptoms. Notably, the total spirituality score was moderately and negatively associated with interpersonal sensitivity (r = −0.52, *p* < 0.01) and paranoid ideation (r = −0.36, *p* < 0.05), with the latter falling in the weak range. Among the spiritual subscales, search for support exhibited a strong negative correlation with interpersonal sensitivity (r = −0.62, *p* < 0.01), while trust in higher guidance was weakly and negatively linked to depression (r = −0.38, *p* < 0.05). Full correlation coefficients are also presented in Table 2.

A multiple linear regression analysis was further performed to examine the extent to which psychological and spiritual variables predicted somatization among mothers of children with T1DM. The model demonstrated excellent fit, with an overall R of 0.924 and R^2^ of 0.854, indicating that 85.4% of the variance in somatization was explained by the predictors. The intercept of the regression model was not statistically significant (B = −0.95, t = −0.87, *p* = 0.385), indicating that the predicted value of somatization when all predictors are set to zero does not differ from zero.

Several psychological variables emerged as significant positive predictors of somatization, including aggression (b = 0.453, *p* < 0.001), paranoid ideation (b = 0.582, *p* < 0.001), depression (b = 0.256, *p* = 0.007), and anxiety (b = 0.288, *p* = 0.011), highlighting the strong association between emotional and cognitive distress and somatic symptomatology. Interestingly, phobic anxiety (b = −0.120, *p* = 0.019) and obsessive–compulsive symptoms (b = −0.370, *p* < 0.001) were negatively associated with somatization, suggesting that these symptoms may not contribute to—or may even mitigate—somatic expression in this context.

Regarding spirituality, trust in higher guidance was a significant negative predictor (b = −0.775, *p* < 0.001), supporting its potential role as a protective factor. Conversely, search for support/access was positively associated with somatization (b = 0.704, *p* < 0.001), potentially reflecting heightened spiritual searching in response to distress. The total spirituality score and the reflection subscale were not statistically significant predictors. Detailed coefficients are provided in Table 3.

## 4. Discussion

The present study examined the relationship between spirituality and psychological functioning in mothers of children with T1DM, with a particular focus on the potential protective role of spirituality in fostering caregiver resilience. Overall, our findings reveal that both psychological (aggression, paranoid ideation, depression, anxiety) and spiritual variables (trust in higher guidance, search for support) play a significant role in predicting somatization in mothers of children with T1DM. Notably, spiritual trust appears to serve as a robust protective factor, while seeking external support is associated with increased somatic symptoms. Collectively, these findings underscore the distinct and influential roles of both psychological and spiritual variables in shaping somatic symptom expression within this caregiving population.

The findings of the present study contribute to a well-established body of literature emphasizing the pervasive psychological impact of caregiving in the context of chronic illness. Caregiver burden is now widely recognized as a systemic and relational phenomenon, with far-reaching effects on family dynamics, marital relationships, and child health outcomes [9,22,23]. Prior studies, such as that by Semerci et al. [19], have demonstrated that religious coping can significantly mitigate levels of caregiver burden, depression, anxiety, and stress among parents of pediatric oncology patients. Similarly, Triana and Sudjatmiko [20] identified religious coping as an effective buffer against the psychological strain associated with caregiving for individuals with schizophrenia. These findings reflect a growing recognition of spirituality as both a personal and communal resource. There is increasing consensus that interventions are most effective when they address the spiritual as well as the psychological needs of caregivers, ideally at both the individual and family system levels [20,24]. Importantly, the experience and expression of spirituality are highly variable across cultures, highlighting the necessity for culturally sensitive frameworks in both research and intervention [5,24].

A unique contribution of the present study is its simultaneous examination of psychological and spiritual predictors of somatization in a Greek sample of mothers caring for children with T1DM. While previous research has largely addressed either psychological or spiritual coping in isolation, this study integrates both domains within a single predictive model, offering a more comprehensive perspective on caregiver adaptation. Additionally, the use of culturally validated instruments ensures the relevance and interpretability of the findings within the Greek context [13,16] while also providing a foundation for cross-cultural comparisons in future research. By demonstrating the differential roles of specific psychological (e.g., aggression, paranoid ideation) and spiritual variables (e.g., trust in higher guidance, search for support) in predicting somatization, the study advances current understanding of how distress is both managed and expressed among mothers facing the chronic illness of a child.

A key finding of the present study is the prominent role of aggression and paranoid ideation as the strongest psychological predictors of somatization. The positive association between aggression and somatic symptoms (b = 0.453, *p* < 0.001) may reflect a tendency for unresolved frustration, irritability, or perceived loss of control to be channeled into physical complaints. This is consistent with theoretical frameworks that conceptualize somatization as an outcome of unprocessed or suppressed emotional distress, particularly in caregiving contexts marked by chronic stress and emotional burden [1]. Similarly, the robust predictive value of paranoid ideation (b = 0.582, *p* < 0.001) suggests that persistent vigilance, mistrust, or feelings of threat—common among individuals facing ongoing uncertainty—may heighten vulnerability to somatic symptomatology. These findings align with the previous literature indicating that maladaptive cognitive patterns and difficulties in emotional processing can intensify both psychological and physical manifestations of distress [2]. Depression and anxiety also predict somatization, though to a lesser extent. These symptoms may compound the emotional burden of caregiving, overwhelming coping mechanisms and increasing somatic complaints [25]. Somatization may also function as a defensive coping strategy, providing a socially acceptable outlet for psychological distress.

Of particular note, the study demonstrates a clear protective role for spiritual trust, whereas the search for support was unexpectedly linked with increased somatic complaints. Trust in higher guidance emerged as a robust negative predictor of somatization (b = −0.775, *p* < 0.001), indicating that mothers who place greater trust in a higher power or spiritual source experience fewer physical symptoms of psychological distress. This finding is well supported by previous research. For instance, Velasco-Durantez et al. [25] found that trust-based spiritual coping was associated with reduced distress and somatic symptoms, particularly when compared to anxious or preoccupied coping styles. Similarly, Musavimoghadam et al. [10] reported that spiritual understanding and a sense of meaning predicted lower psychological distress among mothers of children with special needs. These results suggest that the benefits of spiritual coping depend on how spiritual resources are internalized and accessed, with spiritual trust enhancing emotional regulation and resilience.

In contrast, the positive association between searching for support and somatization (b = 0.704, *p* < 0.001) suggests a more complex or even paradoxical relationship. Rather than reflecting a purely adaptive coping strategy, frequent search for support may indicate ongoing psychological strain or unmet psychosocial needs. This interpretation is consistent with evidence from Velasco-Durantez et al. [25], who observed that individuals with higher levels of anxious preoccupation—often leading to frequent support seeking—also reported increased psychological distress and somatic symptoms. Furthermore, García-Sierra et al. [26] found that, in primary care settings, perceived psychosocial risk (often associated with support-seeking behavior) was linked to psychological distress, though not always directly to somatization. These findings emphasize the importance of distinguishing between adaptive, emotionally supportive coping and support seeking driven by unresolved distress. Interestingly, neither the total spirituality score nor reflective spiritual practices significantly predicted somatization, aligning with previous research that not all aspects of spirituality are equally protective [10]. Instead, specific elements, such as trust and spiritual connectedness, appear more influential in psychological adaptation, suggesting that the quality and internalization of spiritual engagement are crucial for mothers managing chronic caregiving demands.

An unexpected finding of this study was the negative association between obsessive–compulsive symptoms (OCSs), phobic anxiety, and somatization. This may indicate that individuals with higher OCSs or phobic anxiety may express psychological distress through alternative, non-somatic pathways. Prior research supports this interpretation, indicating that in obsessive–compulsive disorder, initial somatic and phobic anxiety symptoms are often replaced by compulsive behaviors as the disorder progresses [27]. Comparatively, generalized anxiety disorder is associated with higher levels of somatization than OCD, highlighting differences in symptom expression and coping mechanisms [28]. Additionally, studies have found no significant difference in OCSs between individuals with high somatization and normative controls, further suggesting that OCSs may serve as an alternative outlet for psychological distress, potentially buffering against somatic symptom development [29].

The present findings have important implications for the psychosocial care of mothers caring for children with chronic illnesses, such as T1DM. First, the identification of aggression and paranoid ideation as strong predictors of somatization highlights the need for interventions that target emotional regulation and maladaptive cognitive patterns. Psychotherapeutic approaches, such as cognitive behavioral therapy (CBT) or acceptance and commitment therapy (ACT), may be particularly effective in helping caregivers recognize, process, and manage intense emotions while also challenging rigid or distress-promoting thought patterns [30]. Equally important is the nuanced role of spirituality revealed in this study. The protective effect of trust in higher guidance suggests that interventions aimed at fostering adaptive spiritual trust—such as meaning-centered psychotherapy or spiritually integrated CBT—could enhance resilience and reduce the physical manifestation of psychological distress. However, the finding that the search for support is associated with increased somatization underscores the necessity of differentiating between effective and reactive coping strategies. Interventions should be designed to help caregivers discern between constructive help seeking and support-seeking patterns that may perpetuate distress or reflect unmet psychosocial needs.

Given the multidimensional nature of spirituality and its variable impact on caregiver well-being, routine spiritual assessment should be integrated into psychosocial care for mothers of chronically ill children. This would enable healthcare professionals to identify both protective spiritual resources and areas of potential vulnerability, allowing for more individualized and holistic intervention planning. Incorporating spirituality into the broader framework of caregiver support may not only improve maternal psychological health but also positively influence family functioning and child health outcomes.

Despite the valuable insights provided by this study, several limitations must be acknowledged. First, the cross-sectional design precludes any causal interpretations regarding the relationships between spirituality, psychological symptoms, and somatization. Longitudinal or prospective studies are needed to clarify the directionality and temporal dynamics of these associations. Second, the reliance on self-report questionnaires may introduce bias related to social desirability or subjective interpretation of items, particularly with respect to sensitive topics, such as psychological distress and spiritual beliefs. Third, the sample was composed exclusively of mothers from a specific cultural and geographic context (Greece), where spirituality and religious identity may hold particular significance; thus, these findings may not generalize to fathers, other caregivers, or populations with different cultural or religious backgrounds.

Several participants also scored zero on the spirituality measures, which may reflect low spiritual identification, secular beliefs, or limitations of the SpREUK instrument in this context. Regarding the linear regression, the non-significant regression intercept (t = −0.87, *p* = 0.385), despite a high R-squared value (0.92), also suggests that the model’s predictive capacity may be less robust than it initially appears. This underlines the importance of cautious interpretation of regression coefficients and related statistics. Furthermore, potential confounding factors, such as socioeconomic status or the severity and duration of the child’s illness, were not fully controlled for and may have influenced the observed relationships. The majority of participants were married and had at least a high school or university education, which may introduce selection bias and limit the applicability of the findings to mothers with lower educational attainment or from less advantaged backgrounds. Although a broad age range was represented for both mothers and children, subgroup analyses based on child age were not feasible due to limited sample sizes, increasing the risk of Type II errors and precluding conclusions regarding developmental differences in caregiving experiences. Moreover, the analysis did not incorporate potentially relevant variables, such as broader social support networks, specific cultural influences, or detailed individual coping skills, all of which could further elucidate the determinants of somatization in this population.

Future research should address current limitations by employing larger, more diverse samples, utilizing longitudinal and mixed methods designs, and including a broader range of contextual and psychosocial variables. It will be important to investigate whether specific aspects of spirituality, such as reflective processing or spiritual trust, moderate the impact of psychological stressors and to clarify the temporal relationship between spirituality and psychological distress. Expanding studies to include fathers and other caregivers can provide a more comprehensive understanding of family dynamics in chronic illness. Intervention studies evaluating the effectiveness of psychosocial and spiritually oriented programs, as well as the use of objective health measures alongside self-report data, are also warranted. Further, research should explore how caregiver psychological and spiritual functioning influences child outcomes, including adjustment and glycemic control, while considering factors such as religious affiliation, socioeconomic status, healthcare access, and community support. Qualitative approaches could offer deeper insights into caregivers’ lived experiences, and long-term studies may identify patterns of resilience or vulnerability. Finally, replicating these findings in secular or religiously diverse and cross-cultural settings will be essential for establishing their generalizability and cultural specificity.

## 5. Conclusions

In summary, this study highlights the complex and multidimensional interplay between psychological and spiritual factors in shaping the health and well-being of mothers caring for children with chronic illnesses. The results highlight the need for integrated approaches to support that address both emotional regulation and spiritual resilience, tailored to the unique contexts and resources of individual caregivers. Developing and implementing comprehensive, multidimensional interventions holds promise for enhancing maternal mental health, strengthening family functioning, and ultimately improving outcomes for children living with chronic health conditions.

## Figures and Tables

**Table 1:** Descriptive statistics of demographic and psychological variables.

Variable	Mean	SD	Min	Max
Mother’s Age	39.62	5.28	18.0	56.0
Number of Children	1.71	0.64	1.0	3.0
Age of Child	8.62	2.89	1.0	17.0
Years of Disease	3.75	2.07	1.0	12.0
Somatization	6.91	6.48	0.0	31.0
Obsessive–Compulsive	9.25	5.98	0.0	32.0
Inter. Sensitivity	6.71	5.38	0.0	30.0
Depression	11.39	8.22	0.0	47.0
Anxiety	7.14	5.53	0.0	29.0
Aggression	3.61	3.72	0.0	22.0
Phobic Anxiety	2.66	3.41	0.0	23.0
Paranoid Ideation	5.59	4.0	0.0	19.0
Psychoticism	4.77	4.28	0.0	25.0
Total Spirituality Score	41.61	16.19	0.0	75.0
Search For Support/Access	11.87	7.3	0.0	25.0
Trust in Higher Guidance/Source	13.33	6.03	0.0	25.0
Reflection	16.42	4.33	0.0	25.0

Notes: SD = standard deviation, Min = minimum, Max = maximum.

**Table 2:** Correlation between spiritual and religious attitudes and psychopathology of the examined sample.

	a	1	2	3	4	5	6	7	8	9	10	11	12
1. Somatization	0.815												
2. Obsessive–Compulsive	0.858	0.75 ***											
3. Inter. Sensitivity	0.907	0.76 ***	0.76 ***										
4. Depression	0.895	0.76 ***	0.87 ***	0.72 ***									
5. Anxiety	0.904	0.76 ***	0.82 ***	0.72 ***	0.79 ***								
6. Aggression	0.911	0.80 ***	0.74 ***	0.83 ***	0.68 **	0.68 **							
7. Phobic Anxiety	0.865	0.65 **	0.69 **	0.63 **	0.70 ***	0.79 ***	0.59 **						
8. Paranoid Ideation	0.895	0.78 ***	0.77 ***	0.86 ***	0.74 ***	0.75 ***	0.74 ***	0.57 **					
9. Psychoticism	0.897	0.68 **	0.80 ***	0.62 **	0.78 ***	0.84 ***	0.61 **	0.78 ***	0.68 **				
10. Total Spirituality Score	0.914	−0.17	−0.32 *	−0.52 **	−0.26	−0.20	−0.39 *	−0.16	−0.36 *	0.19			
11. Search For Support/Access	0.958	−0.12	−0.24	−0.62 **	−0.14	0.06	−0.38 *	0.22	−0.46 *	0.22	0.93 ***		
12. Trust in Higher Guidance/Source	0.707	−0.17	−0.34 *	−0.27	−0.38 *	0.02	−0.25	0.09	−0.18	0.17	0.89 ***	0.72 ***	
13. Reflection	0.795	−0.18	−0.34 *	−0.46 *	−0.23	−0.15	−0.44 *	0.08	−0.27	0.08	0.87 ***	0.71 ***	0.72 ***

Notes: a = Cronbach’s alpha, *** *p* < 0.001, ** *p* < 0.01, * *p* < 0.05. Correlations above 0.70 were considered strong, those between 0.40 and 0.69 moderate, and those below 0.40 weak.

**Table 3:** Multiple linear regression: predicting somatization from psychological and spiritual variables.

Variable	B	Beta (Standardized)	SE	t	*p*
Const.	−0.946	-	1.0863	−0.8717	0.385
Obsessive–Compulsive	−0.335	−0.370	0.0901	−3.7185	0.000
Inter. Sensitivity	0.025	−0.010	0.0996	0.2519	0.801
Depression	0.185	0.256	0.0675	2.7423	0.007
Anxiety	0.282	0.288	0.1099	2.5655	0.011
Aggression	0.777	0.453	0.1514	5.1343	0.000
Phobic Anxiety	−0.354	−0.120	0.1498	−2.3607	0.019
Paranoid Ideation	0.886	0.582	0.1234	7.1851	0.000
Psychoticism	−0.041	−0.026	0.1011	−0.4033	0.687
Total Spirituality Score	0.006	0.066	0.012	0.4719	0.637
Search For Support/Access	0.457	0.704	0.0773	5.9232	0.000
Trust in Higher Guidance/Source	−0.560	−0.775	0.0808	−6.9277	0.000
Reflection	0.108	0.138	0.067	1.6148	0.108

Notes: B = unstandardized regression coefficient; SE = standard error; t = t-statistic; *p* = *p*-value. Mothers’ age, education, and marital status were considered as potential moderators.

## Data Availability

The data presented in this study are available upon request from the corresponding author.

## Supplementary Materials

The following supporting information can be downloaded at https://www.mdpi.com/article/10.3390/healthcare13121439/s1, Table S1: Percentiles (25th, 50th, and 75th) for SCL-90 and SpREUK Subscales.
