# Supplementary material for: Psychological Characteristics of Mothers of Children with Chronic Illnesses: A Focus on Type 1 Diabetes Mellitus
Source: Healthcare (Basel). 2025 Jun 16;13(12):1439. doi: 10.3390/healthcare13121439 (PMC12192864; doi:10.3390/healthcare13121439)
Supplement: Supplementary file 1 [file healthcare-13-01439-s001.zip › healthcare-3644620-supplementary.pdf]

Supplementary Table S1. Percentiles (25th, 50th, and 75th) for SCL-90 and SpREUK Subscales

Subscale	25th Percentile	Median (50th)	75th Percentile
Somatization	5	5	8
Obsessive-Compulsive	7	9	10
Interpersonal Sensitivity	2	7	8
Depression	6	12	14
Anxiety	3	6	10
Aggression	1	2	5
Phobic Anxiety	0	2	4
Paranoid Ideation	4	4	8
Psychoticism	1	5	6
Total Spirituality Score	36	43	50
Search For Support/Access	7	12	17
Trust in Higher Guidance/Source	9	13	17
Reflection	13	17	19
